# Application of low‐dose CT to the creation of 3D‐printed kidney and perinephric tissue models for laparoscopic nephrectomy

**DOI:** 10.1002/cam4.3851

**Published:** 2021-04-02

**Authors:** Guan Li, Jie Dong, Zhiqiang Cao, Jinbao Wang, Dongbing Cao, Xin Zhang, Longjiang Zhang, Guangming Lu

**Affiliations:** ^1^ Department of Radiology Jinling Hospital Medical School of Nanjing University Nanjing China; ^2^ Department of Urology Jinling Hospital Medical School of Nanjing University Nanjing China; ^3^ Department of Urology General Hospital of Northern Theater Command Shenyang China; ^4^ Department of Radiology General Hospital of Northern Theater Command Shenyang China; ^5^ Department of Urology Cancer Hospital of China Medical University Shenyang China; ^6^ Department of Radiology The First Affiliated Hospital of China Medical University Shenyang China

**Keywords:** 3D printing, adherent perinephric fat, computed tomography, low‐dose, renal tumor

## Abstract

**Purpose:**

The aim of this study was to explore the feasibility of 3D printing of kidney and perinephric fat based on low‐dose CT technology.

**Patients and Methods:**

A total of 184 patients with stage T1 complex renal tumors who underwent laparoscopic nephrectomy were prospectively enrolled and divided into three groups: group A (conventional dose kidney and perinephric fat 3D printing group, *n* = 62), group B (low‐dose kidney and perinephric fat 3D printing, *n* = 64), and group C (conventional dose merely kidney 3D printing group, *n* = 58). The effective dose (ED), signal‐to‐noise ratio (SNR), and contrast‐to‐noise ratio (CNR) were determined. The 3D printing quality was evaluated using a 4‐point scale, and interobserver agreement was assessed using the intraclass correlation coefficient (ICC).

**Results:**

The ED of group B was lower than that of group A, with a decrease of 55.1%. The subjective scores of 3D printing quality in all groups were 3 or 4 points. The interobserver agreement among the three observers in 3D printing quality was good (ICC = 0.84–0.92). The perioperative indexes showed that operation time (OT), warm ischemia time (WIT), estimated blood loss (EBL), and laparoscopic partial nephrectomy (LPN) conversion to laparoscopic radical nephrectomy (LRN) in groups A or B were significantly less than those in group C. LPN was more frequent in group A and group B than in group C (all *p* < 0.017). There were no significant differences in perioperative indexes between group A and group B (all *p* > 0.017).

**Conclusion:**

Low‐dose CT technology can be effectively applied to 3D printing of kidney and perinephric fat and reduce the patient's radiation dose without compromising 3D printing quality. 3D printing of kidney and perinephric fat can significantly increase the success rate of LPN and decrease OT, WIT, and EBL.

## Graphical abstract

Low‐dose CT technology can effective applied to 3D printing of kidney and perinephric fat, reduced patient’s radiation dose without compromising 3D printing quality. 3D printing of kidney and perinephric fat can significantly increase the success rate of LPN and decrease OT, WIT and EBL.

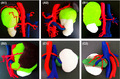

## 1 INTRODUCTION

With the rapid development of three‐dimensional (3D) printing technology, 3D printing has been widely used in the medical field in contexts such as neurosurgery, hepatobiliary surgery, orthopedics, plastic surgery, cardiac surgery, and urology. Kidney 3D printing can show the kidney, renal tumor, renal artery, and renal vein and the anatomical relationships of the surrounding tissue structure.^1^ Kidney 3D printing is conducive to the formulation of surgical plans, surgical risk assessment, simulation of surgical processes, and doctor–patient communication.^2^ Precision medicine has become a hot spot in the medical field. Strict requirements have been proposed for kidney 3D printing. When adherent perinephric fat (APF) occurs, it can lead to difficulty during partial nephrectomy (PN); the operation time is prolonged, the probability of hemorrhage and renal fibrous membrane tears is increased, and patients are even forced to switch to radical nephrectomy (RN).^3^, ^4^ Hence, 3D printing of APF is particularly important. Through the 3D printing of APF, 3D printing is more individualized.

The original CT Digital Imaging and Communications in Medicine (DICOM) files provided by CT scanners are often used as the database for 3D printing.^5^ To obtain better quality 3D‐printed models, the original source images should be of high quality, which can lead to a higher radiation dose. However, excessive CT radiation doses can lead to an increased lifetime risk of cancer.^6^, ^7^ In recent years, low‐dose CT has been widely used in clinical practice. However, whether low‐dose CT technology can be used in 3D printing is rarely reported. The aim of this study was to explore the feasibility of creating 3D‐printed kidney and perinephric fat models using low‐dose CT images when compared to the standard renal CT protocol.

## 2 PATIENTS AND METHODS

### 2.1 Study design and population

The study was approved by the Ethics Committee of the Jinling Hospital, Nanjing Medical University, and all patients provided informed consent before the examination. Patients (*n* = 184; 125 males and 59 females, with an age range of 32 ~ 87 years and a median age of 59.5 ± 13.3 years) with renal tumors (RENAL nephrometry score ≥7)^8^ who were recommended to undergo laparoscopic partial nephrectomy (LPN) or laparoscopic radical nephrectomy (LRN) at our institution were reviewed prospectively between October 2018 and August 2020. A total of 184 patients were randomly divided into three groups, namely, group A (conventional dose kidney and perinephric fat 3D printing group, *n* = 62), group B (low‐dose kidney and perinephric fat 3D printing, *n* = 64), and group C (conventional dose kidney alone 3D printing group, *n* = 58). All operations were performed by three urologists (J.D., Z.Q. C., and D.B. C.). The perioperative indicators of patients were recorded according to surgical records and postoperative follow‐up.

### 2.2 Inclusion and exclusion criteria

The inclusion criteria were as follows: (I) renal artery CTA examination; (II) stage T1 renal tumor; (III) RENAL nephrometry score ≥7; and (IV) Mayo adhesive probability (MAP) score ≥3. The exclusion criteria were as follows: (I) CT contrast agent allergy; (II) severe cardiovascular and cerebrovascular diseases; (III) severe kidney dysfunction; (IV) severe renal artery stenosis or occlusion; and (V) renal vascular stent therapy.

### 2.3 CT scanning protocol

All patients underwent imaging on a multidetector CT scanner (Discovery CT750 HD; GE Healthcare). The CT scanning range was from the bilateral adrenal level to the bilateral lower pole. The scan parameters for groups A and C were as follows: tube voltage 120 kVp, tube current 400 mA, and reconstruction using the standard filtered back projection (FBP) algorithm. For group B, the scan parameters were as follows: tube voltage 80 kVp, tube current 400 mA, reconstruction using the 50% adaptive statistical iterative reconstruction (ASiR) algorithm, and noise index (NI) 11 HU.

Other scanning parameters were applied as follows: 64 detectors with a 0.625 mm section thickness; beam collimation: 40 mm; rotation time: 0.5 s; pitch: 0.984:1; image matrix: 512 × 512; field of view: 250 mm; noise index: 11.0; scan slice thickness: 5.00 mm; and reconstruction slice thickness: 0.625 mm.

### 2.4 Contrast medium injection scheme

All patients were injected with 350 mgI/mL contrast medium (Omnipaque 350, GE Healthcare) through the median elbow vein. The contrast medium dosage was approximately 45 ~ 75 ml (0.9 ~ 1.0 ml/kg), with a flow rate of 5 ml/s, followed by a 30 ml saline flush at the same flow rate. For patients with large body weights, the dosage of contrast (70 ~ 95 ml) was increased appropriately. The contrast medium was injected using a high‐pressure syringe (Urich, Medical). Automatic bolus tracking was used, with a trigger threshold for the abdominal aorta of 150 Hounsfield units (HU).

### 2.5 Radiation dose

The volumetric CT dose index (CTDIvol) and dose‐length product (DLP) provided by the CT scanner were recorded. The effective radiation dose was calculated according to the formula ED = DLP × K, where K was the conversion factor of 0.015 mSv/(mGy·cm).^9^ Because the CT scanning conditions and contrast medium dosage were consistent between group A and group C, we selected group A as the representative for evaluation.

### 2.6 3D modeling and printing

All patients with CT DICOM files were input into 3D printing software (Materialize Mimics 18.0.0.524) to undergo 3D reconstruction. The automatic segmentation function of 3D printing software was used to segment different tissue types. The automatic segmentation function of the 3D printing software was used to segment different tissue types, such as kidney, tumor, blood vessel, liver, spleen, and bone. The principle of the 3D printing software automatic segmentation function is based on the CT fixed thresholds; for example, the CT threshold of soft tissue was 40 ~ 60 Hu, the CT threshold of fat tissue was −10~‐30 Hu, and the CT threshold of bone tissue was 1000 Hu. For some tissues that could not be recognized automatically or for which the segmentation effect was not good, we used the method of manually drawing regions of interest (ROIs) for segmentation. They were the same across patients for each scan. There were no significant differences in mean HUs between group A, group B, and group C, but the mean HUs in normal perinephric fat and APF were different. We selected a radiologist (J.B. W.) with 15 years of work experience to confirm the results. By manually removing unconnected structures, we reserved only the kidney, tumor, renal arteries, renal veins, abdominal aorta, and APF area. We chose different colors to tag the different target organs, tissues, and tumors. All target organs, tissues, and tumors that were expanded were triangulated and smoothed in turn and saved separately as standard tessellation language (STL) files. Then, all STL files were imported into the 3D modeling software (Materialize 3‐matic, 10.0.0.212). Finally, a 3D printing rapid prototyping printer (MakerBot Replicator Z18) was used to finish 3D printing. We used fused deposition modeling (FDM) technology and acrylonitrile butadiene styrene (ABS) thermoplastic material to finish the 3D printing. The time to complete each 3D printing model was approximately 22–24 h, and the 3D printing cost was approximately RMB 2,000–3,000.

### 2.7 Quantitative evaluation

The goal of quantitative evaluation was to achieve good segmentation based on a high contrast‐noise ratio (CNR) and signal‐to‐noise ratio (SNR). The measurement of objective quality for groups A and B was performed by two radiologists (J.B. W. and X. Z.), with 15 and 20 years of experience in image postprocessing. On the GE AW4.6 workstation, the region of interest (ROI) was selected to ensure the same target substance. The size of the ROI was defined as 50% larger than the area of the vascular lumen or 100 mm^2^ (other parts except blood vessels), avoiding the vascular wall and vascular calcification. The location of the ROI was selected to measure the CT value at the same level of the renal artery (RA) (approximately 1.0 cm from the beginning of the renal artery), abdominal aorta (AA), erector spinae (ES), and air in front of the anterior abdominal wall. The standard deviation (SD) of air in front of anterior abdominal wall CT values was defined as image noise (IN). Based on the above measurements, the CNR and SNR were obtained using the following formula: CNR = (CT_RA_–CT_ES_) / SD_air_ and SNR = CT_RA_ / SD_air_.

### 2.8 Qualitative evaluation

The 3D printing products of groups A and B were reviewed and independently scored by three urologists (J.D., Z.Q. C., and D.B. C., with 18, 30, and 9 years of operative experience, respectively) who were blinded to the scanning protocols. At present, there is no authoritative organization to formulate evaluation standards for 3D printing quality. We referred to the related literature on 3D printing quality and the opinions of surgeons to formulate the 3D printing quality score.^10^

All 3D printing products quality were evaluated on a 4‐point scale as follows: 4 (excellent), the 3D printing structure of the kidney, tumor, blood vessels is intact and vivid, and the size, shape, and location of the tumor and the area of APF are accurate; 3 (good), the 3D printing structure of the kidney, tumor, blood vessels is complete and clear, and the size, shape, and location of the tumor and the area of APF are correct; 2 (poor), the 3D printing structure of the kidney, tumor, blood vessels is partially missing and coarse, and the size, shape, and location of the mass and the area of APF are blurred; and 1 (low), the 3D printing structure of the kidney, tumors, blood vessels is large partially missing and unclear, and the size, shape, and location of the mass and the area of APF are inaccurate. It was considered that 3D printing products with 3 points or more were satisfactory for urologists. In case of interobserver disagreement, the final decisions were reached by consensus.

### 2.9 Statistical analysis

SPSS version 17.0 (SPSS Inc.) was used for statistical analysis. Quantitative data are shown as the mean±standard deviation (SD). Categorical variables were described as frequencies or percentages. Mean comparisons between two independent samples were performed with Student's *t*‐test. The hierarchical data of the two groups were analyzed by the Wilcoxon test. The Kruskal–Wallis test was used to compare several independent samples. The counting data of the two groups were examined by the χ^2^ test. The Pearson chi‐squared test was used to test the correlation between the two groups. Interobserver agreement was assessed using the intraclass correlation coefficient (ICC). A *p* value of <0.05 was considered statistically significant.

## 3 RESULTS

### 3.1 Patient demographics

Table 1 summarizes the patient demographics. Comparisons of age, height, weight, sex, body mass index (BMI), RENAL score, and MAP score between group A, group B, and group C showed no significant differences (all *p* > 0.05).

**TABLE 1:** Patient demographics of group A, group B, and group C

Variable	Group A (*n* = 62)	Group B (*n* = 64)	Group C (*n* = 58)
Mean age (years)	58.1 ± 13.4	57.6 ± 12.6	55.8 ± 13.8
Height (cm)	169.5 ± 8.2	167.2 ± 9.4	172.3 ± 10.6
Weight (kg)	76.3 ± 11.8	74.4 ± 12.3	81.2 ± 13.1
Sex (%)			
Male	42 (68%)	39 (61%)	34 (59%)
Female	20 (32%)	25 (39%)	24 (41%)
BMI (kg/m^2^)	26.9 ± 4.2	26.5 ± 5.4	27.1 ± 5.7
MAP score	3.6 ± 2.1	3.9 ± 1.9	4.0 ± 2.2
RENAL score	8.6 ± 2.2	8.1 ± 1.3	8.3 ± 2.1

### 3.2 Quantitative analysis

**FIGURE 1:**
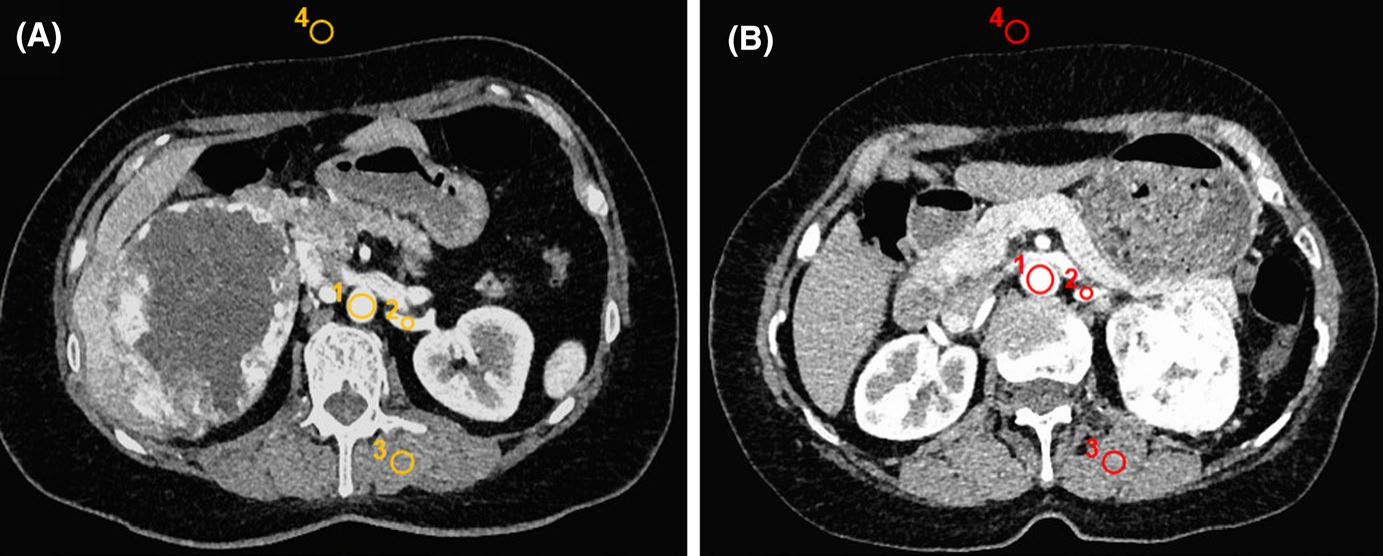
Region of interest selected between Groups (A) and (B). (A) CT DICOM image using the group (A) protocol in a 46‐year‐old male. (B) CT DICOM image using the group (B) protocol in a 52‐year‐old male. ROI_1_: abdominal aorta (AA); ROI_2_: renal artery (RA); ROI_3_: erector spinae (ES); ROI_4_: air

Table 2 shows the comparative results of relevant CT values, IN, CNR, and SNR between groups A and B. There were no significant differences in the CT values of air, IN, CNR, and SNR between group A and group B (−987.3 ± 5.9 vs. −988.9 ± 6.6 HU, 11.6 ± 5.9 vs. 13.7 ± 5.2, 21.9 ± 3.1 vs. 23.2 ± 3.6, 27.4 ± 3.8 vs. 28.7 ± 3.2, all *p* >.05). The CT values of RA, AA, and ES in group A (313.9 ± 52.6 HU, 338.0 ± 55.1 HU and 58.5 ± 4.5 HU) were significantly lower than those in group B (406.6 ± 57.3 HU, 431.2 ± 59.5 HU, and 90.6 ± 7.1 HU) (all *p* <0.001). (Figure 1).

**TABLE 2:** Quantitative analysis of images in group A and group B

Index	Group A (*n* = 62)	Group B (*n* = 64)	*p* value
RA (HU)	313.9 ± 52.6	406.6 ± 57.3	<0.001
AA (HU)	338.0 ± 55.1	431.2 ± 59.5	<0.001
ES (HU)	58.5 ± 4.5	80.6 ± 7.1	<0.001
Air (HU)	−987.3 ± 5.9	−988.9 ± 6.6	0.204
IN	11.6 ± 5.9	13.7 ± 5.2	0.062
CNR	21.9 ± 3.1	23.2 ± 3.6	0.056
SNR	27.4 ± 3.8	28.7 ± 3.2	0.067

Abbreviations: RA, renal artery; AA, abdominal aorta; ES, erector spinae; IN, image noise; CNR, contrast‐noise ratio; SNR, signal‐to‐noise ratio.

### 3.3 Radiation dose measurement results

The CTDI_vol_, DLP, and ED values for group A were significantly higher than those for group B (13.7 ± 1.4 vs. 5.0 ± 0.6 mGy for CTDI_vol_; 315.6 ± 23.1 vs. 132.8 ± 11.2 mGy‐cm for DLP; 4.9 ± 0.6 vs. 2.2 ± 0.7 mSv for ED; all *p* < 0.001; Table 3). Compared with group A, the CTDIvol, DLP, and ED in group B decreased by 63.5%, 57.9%, and 55.1%, respectively. These results suggest that the protocol in group B can significantly reduce the CT radiation dose.

**TABLE 3:** Radiation dose analysis of images in group A and group B

Index	Group A (*n* = 62)	Group B (*n* = 64)	*p* value
CTDI_vol_ (mGy)	13.7 ± 1.4	5.0 ± 0.6	<0.001
DLP (mGy‐cm)	315.6 ± 23.1	132.8 ± 11.2	<0.001
ED (mSv)	4.9 ± 0.6	2.2 ± 0.7	<0.001

### 3.4 Qualitative analysis

**FIGURE 2:**
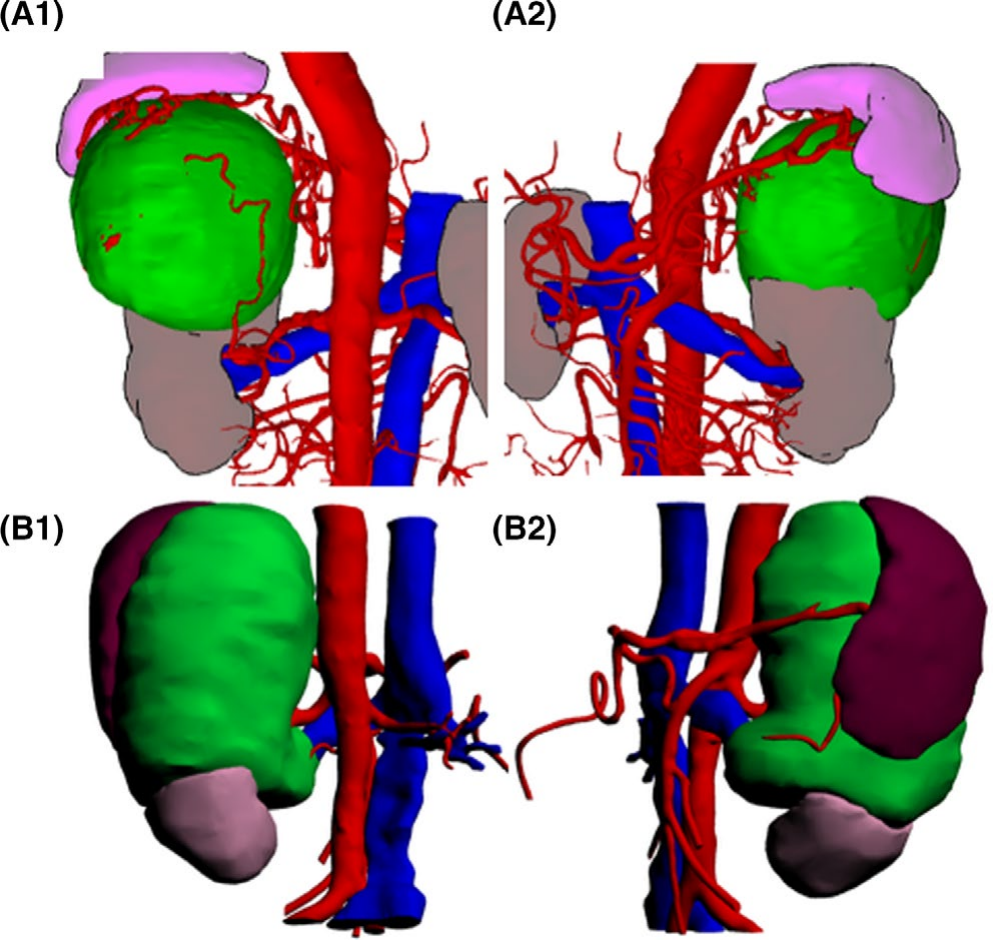
3D modeling results of kidney and APF in groups (A) and (B) (A1‐2: male, 46 years, with left kidney tumor, adopt group (A) protocol, transparency is 60%; B1‐2: male, 52 years, with left kidney tumor, adopt group (B) protocol, transparency is 20%; gray: kidney; green: renal tumor; purple or brown: APF; red: artery; blue: vein)

**FIGURE 3:**
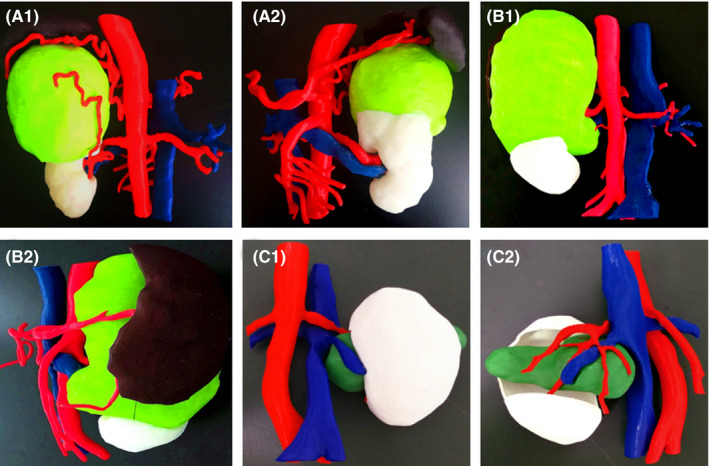
3D printing results of kidney and APF in groups (A) and (B) (A1‐2: male, 46 years, with left kidney tumor, adopt group (A) protocol; B1‐2: male, 52 years, with left kidney tumor, adopt group (B) protocol; C1‐2: male, 65 years, with left kidney tumor, kidney 3D printing without APF; green: renal tumor; white: kidney; brown: APF; red: artery; blue: vein)

Table 4 shows the results of 3D printing quality scores for 184 patients from group A, group B, and group C. After Kruskal–Wallis test analysis, subjective results of 3D printing quality revealed no significant difference between group A, group B, and group C (*p* = 0.702). Furthermore, the interobserver agreement between the three observers in 3D printing quality was good (ICC = 0.84–0.92) (Figures 2, 3).

**TABLE 4:** Subjective scores of 3D printing quality for group A, group B, and group C

Groups	1 score	2 score	3 score	4 score	Total (case)	*p* value
Group A	0	0	27	35	62	0.702
Group B	0	0	25	39	64	0.702
Group C	0	0	27	31	58	

### 3.5 Perioperative outcomes

Table 5 summarizes the perioperative outcomes in the cohort. The results showed that the indexes of operation time (OT), warm ischemia time (WIT), estimated blood loss (EBL), LPN conversion to LRN and underwent LPN were significantly different among groups A, B, and C (all *p* < 0.05). The OT, WIT, EBL, and LPN conversion to LRN of group A or group B were less than those of group C (all *p* < 0.017). There were more patients who underwent LPN in group A or group B than in group C (all *p* < 0.017). There were no significant differences in perioperative outcomes between group A and group B (all *p* > 0.017).

**TABLE 5:** Perioperative outcomes of group A, group B, and group C

Variable	Group A (*n* = 62)	Group B (*n* = 64)	Group C (*n* = 58)
OT, min	95.2 ± 15.7	90.3 ± 16.4	105.2 ± 18.6^a^, ^b^
WIT, min	21.3 ± 6.8	20.9 ± 7.1	25.3 ± 8.2^a^, ^b^
EBL, mL	45.9 ± 11.6	46.2 ± 9.4	57.9 ± 10.1^a^, ^b^
LPN conversion to LRN, cases	5 (8%)	6 (9%)	17 (24%)^a^, ^b^
Hospital stay, days	7.1 ± 1.7	6.9 ± 2.4	7.5 ± 1.8
Complication, cases	5 (8%)	4 (6%)	5 (8%)
Underwent LPN, No. (%)	53 (69%)	54 (63%)	34 (55%)^a^, ^b^
T1a stage, No. (%)	47 (76%)	50 (78%)	46 (79%)
T1b stage, No. (%)	15 (24%)	14 (22%)	12 (21%)

Note

*P* value after Bonferroni correction for multiple comparisons (*p* = 0.05/3 ≈ 0.017). Group A vs. Group B, *p* > 0.017

Abbreviations: OT, operative time; WIT, warm ischemia time; EBL, estimated blood loss; LPN, laparoscopic partial nephrectomy; LRN, laparoscopic radical nephrectomy.

^a^ Group C vs. Group A, *p* < 0.017;

^b^ Group C vs. Group B, *p* < 0.017

## 4 DISCUSSION

With the widespread application of 3D printing technology in the clinic, however, very few people pay attention to the radiation dose issue caused by 3D printing. At present, there are many methods to reduce the CT radiation dose. However, these methods are rarely reported in 3D printing. In the present study, we used a low‐dose CT protocol (80 kVp, 400 mA, and 50% ASiR) for 3D printing and found that the radiation dose was reduced by 55.1% compared with that of the conventional CT protocol (120 kVp, 400 mA, and FBP). A low‐dose CT protocol was able to be produced that can meet the clinical requirements of 3D printing. Furthermore, we have confirmed that compared to conventional kidney 3D printing, 3D printing of kidney and perinephric fat can effectively improve the success rate of LPN and significantly reduce OT, WIT, EBL, and LPN conversion to LRN.

In recent years, renal computed tomography angiography (CTA) has become the routine examination before surgery.^11^ However, traditional renal CTA is a three‐dimensional display on two‐dimensional CT films, which challenges the operator's sense of spatial logic and still has some limitations in the display of some details. At the same time, it is far less accurate, flexible, and vivid than the 3D printing model. Previous studies have shown that the radiation dose of renal CTA is lower than that of renal dynamic contrast‐enhanced CT scanning.^12^, ^13^ Therefore, we chose renal CTA data to finish the 3D printing of renal tumors and APFs. At the same time, we adopted a low‐dose renal CTA scanning protocol to further reduce the radiation dose of 3D printing. At present, the methods for reducing the CT radiation dose include reducing the tube voltage, reducing the tube current, shortening the scanning time, and applying iterative algorithms.^14^, ^15^ Liu et al.^12^ proved that low‐dose CT scanning technology (100 kVp) in renal CTA examination can significantly decrease ED, by 37.24%. However, we chose 80 kVp in the present study, further reduced the tube voltage, and decreased ED by approximately 50.54%. Trattner S et al.^16^ proved that the radiation dose is proportional to the square of the tube voltage. Therefore, a decrease in the tube voltage can greatly reduce the radiation dose. Group B had higher CT attenuation than group A because lowering the tube voltage can significantly increase CT attenuation.^17^ When reducing the tube voltage, the IN can be increased, and the SNR or CNR can be decreased.^18^ However, our study showed that IN, SNR, and CNR were not significantly different between groups A and B. This is a result of using the ASiR (GE Healthcare, Waukesha, WI) algorithm. ASiR is based on the mathematical model and statistical iteration of CT data, and it can effectively decrease image noise and improve SNR and CNR.^19^ Li et al.^20^ reported that ASiR can provide clinically acceptable image quality with an estimated dose reduction in the range of 40%~60%.

In 2014, Davidiuk et al.^21^ first proposed the impact of APF on partial nephrectomy and established a CT image‐based MAP score to predict the possibility of APF before surgery. Dariane C et al.^22^ reported that the presence of APF can increase the complexity of surgery, operative time, and subsequent complications. Therefore, this study is the first attempt to undertake APF 3D printing. Because the area of APF appears to have different extents of perinephric fat stranding and fiber bars of varying thickness on CT DICOM data, we can set the relevant HU threshold according to the changes in perinephric fat density and achieve 3D printing of APF. Relevant studies have shown that 3D printing‐assisted LPN can shorten the operation time and the warm ischemic time and reduce intraoperative blood loss.^23^, ^24^, ^25^ These results are consistent with our study. However, these 3D printing models have not demonstrated the occurrence of APF. Therefore, we concluded that the OT, WIT, and EBL of the 3D‐printed APF group were significantly smaller than those of the 3D‐printed group without APF displays.

The reasons for OT shortening in the kidney and perinephric fat 3D printing group can be analyzed as follows: (I) The preoperative application of the kidney and perinephric fat 3D printing model helps the surgeons correctly formulate the operation plan. When 3D printing shows that the APF area has a large range and is closely related to kidney tumors, forced separation can lead to renal fibrous membrane tears, increased intraoperative blood loss, and failure of postoperative sutures, so we chose LRN directly to avoid the increase in OT from LPN conversion to LRN. (II) The surgeons can fully simulate the operation process before surgery, so that practice is perfect. (III) This helps surgeons locate renal tumors quickly and accurately. WIT shortening can be analyzed as follows: by adopting 3D printing technology, surgeons can accurately identify the responsible vessel of the renal tumor and perform direct clamping, avoiding clamping of the main renal artery, and achieving zero ischemia (WIT=0 min). The reasons for the decrease in EBL can be summarized as follows: the application of a 3D printing model can accurately identify the responsible vessel of the renal tumor and accessory renal artery by directly clamping the responsible vessel and accessory renal artery, avoiding misclamping, and thus reducing EBL.

This study had several limitations. First, the study sample size is very small. Future studies with larger sample sizes are needed to corroborate our findings. Second, we did not adopt a different percentage ASiR algorithm to compare the quality of 3D printing. Third, this study lacks subjective scores of 3D printing quality from surgeons of different seniority. Fourth, we did not evaluate the virtual 3D design results, and there were no quantitative estimates of agreement or evaluations of segmentation accuracy. Finally, the printing of physical properties such as softness and hardness, via 3D printing has not yet been carried out. These limitations will be further discussed in future research.

## 5 CONCLUSIONS

In conclusion, low‐dose CT technology can be effectively applied to 3D printing, reducing ED without compromising 3D printing quality. 3D printing of renal tumors and APF can significantly increase the success rate of LPN, shorten OT and WIT, and reduce EBL.

## CONFLICT OF INTEREST

The authors declare that they have no conflict of interest.

## Data Availability

The data that support the findings of this study are available from the corresponding author upon reasonable request.
